# Simplified Mutual Inductance Calculation of Planar Spiral Coil for Wireless Power Applications

**DOI:** 10.3390/s22041537

**Published:** 2022-02-16

**Authors:** Iftikhar Hussain, Dong-Kyun Woo

**Affiliations:** Department of Electrical Engineering, Yeungnam University, Gyeongsan 38541, Korea; iftikhar.razai@gmail.com

**Keywords:** litz wire, lateral misalignment, magnetic field distribution, mutual inductance, Neumann integral formula, planar spiral coil

## Abstract

In this paper, a simplified method for the calculation of a mutual inductance of the planar spiral coil, motivated from the Archimedean spiral, is presented. This method is derived by solving Neumann’s integral formula in a cylindrical coordinate system, and a numerical tool is used to determine the value of mutual inductance. This approach can calculate the mutual inductances accurately at various coaxial and non-coaxial distances for different coil geometries. The calculation result is compared with the 3D finite element analyses to verify its accuracy, which shows good consistency. Furthermore, to confirm it experimentally, Litz wire is used to fabricate the sample spiral coils. Finally, the comparison of a simplified method is also studied relative to the coupling coefficient. The accuracy of the calculation results with the simulation and the measurement results makes it a good candidate to apply it in wireless power applications.

## 1. Introduction

Planar spiral coils have been widely adopted in many electromagnetic applications ranging from low power such as mobile phones, electric toothbrushes, and biomedical implants [1,2,3,4,5,6,7,8,9,10] to the high-power drone charging systems and electric vehicles. The wireless power transfer (WPT) method was employed to transfer power between these coils [11,12,13,14,15]. WPT consists of a primary coil and a secondary coil. Power is transferred between them by magnetic coupling. To design a coil or calculate the performance related to power, such as voltage gain, efficiency, output power, and transfer distance, determining mutual inductance (*M*) is necessary. In the last few decades, much work has been done to estimate the *M* between coils. Maxwell derived the *M* equation between two coaxial circular filamentary coils from the perspective of energy using a complete elliptic integral [16,17]. Another equation is obtained using the magnetic vector potential approach [18]. However, many applications require a spiral coil instead of a circular coil for higher power transfer and coupling coefficient. The Neumann integral formula was adopted to estimate *M* between two circular filaments carrying a constant current. *M* between spiral coils was predicted by assuming each spiral coil as a group of concentric circular coils. The self-inductances of each circular coil and mutual inductances between them are calculated to obtain the self-inductance of the spiral coil. Finally, the mutual inductance is computed by calculating the inductance between the two spiral coils [19,20]. However, this method needs a lot of calculations. Furthermore, there is always an inconsistency between the calculated result of mutual inductance and simulation and measurement results due to the supposition of the spiral coils as a collection of the circular coils. This problem is solved by employing the Archimedean spiral coil equation considering coil helicity [21]. This method has used a rectangular coordinate system to find the parameters of the Neumann integral formula for mutual inductance. As a result, the derivation steps and the number of parameters in the final *M* equation increase. Furthermore, a separate calculation of each lower and upper limit of a double integral is required. Thus, the *M* equation becomes more complex and needs more time to calculate the *M*. Therefore, a more simplified and fast method is necessary to estimate the *M* accurately.

The finite element method (FEM) using Maxwell 3-D electromagnetic software is one of the most suitable methods to compute mutual inductances; however, it requires a long computational time and a high-speed computer [22].

In this paper, a more simplified form of mutual inductance equation between two circular planar spiral coils is calculated in a cylindrical coordinate using Neumann’ integral formula. Solving *M* in a cylindrical coordinate system significantly reduces the derivation steps and the calculation complexity.

In addition, compared to the conventional method, which required the calculation of each upper and lower limit of the double integral of the final *M* equation, our approach simplified it, starting from 0 to 2π*N*, for all types of the circular spiral coil. Hence, a more simplified *M* equation is obtained, which can be solved by a numerical tool, such as Matlab. Moreover, a simplified *M* equation under lateral misalignment is also calculated. The simplified mutual inductance result is verified by simulation and experiment results. Thus, the comparison of the experiment result with the simulation and calculation result confirms the accuracy of our equation.

The rest of the paper is organized as below: Section 2 describes the derivation of the mutual inductance of planar spiral coil under perfectly aligned and lateral misalignment. The verification of the calculation result with the simulation result using ANSYS Maxwell 15 is discussed in Section 3. Section 4 is the detailed study of experimental verification of calculation and simulation results and is divided into many subsections and sub-subsections. The selection of the wire for the spiral coil and its measurement are determined in Section 4.1 while mutual inductance measurement method is described in Section 4.2. Section 4.3 express the comparison of calculation, simulation, and measurement results for different coil configurations under the coaxial case, and the errors between them are calculated. The effect of mutual inductance as a function of lateral misalignment for different coil configurations is represented in Section 4.4. The variation of calculation results relative to measurement and simulation are also computed in this subsection. Section 4.5 verify the calculation result with the coupling coefficient formula. 

## 2. Mutual Inductance Equation Derivation

### 2.1. Two Perfect Aligned the Planar Spiral Coil

**Figure 1:**
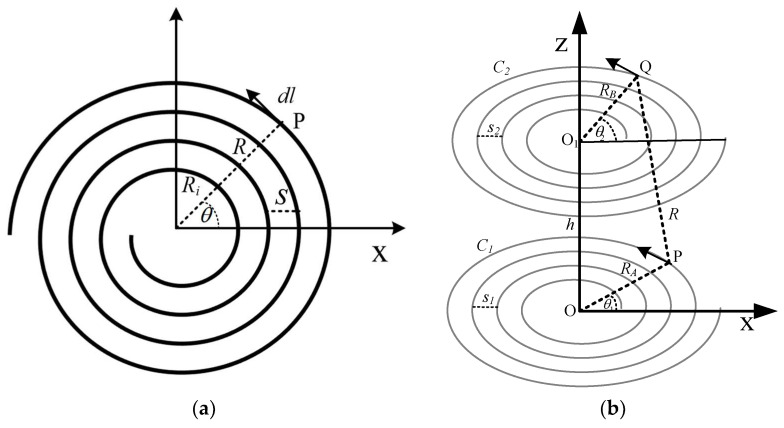
(**a**) Planar spiral coil. (**b**) Aligned circular spiral coil.

The planar spiral and its structural parameters are expressed in Figure 1a. To overcome the skin effect, assume a constant current flows into the coil. Mutual inductance depends on the geometric factors of the coil and the respective distance. Initially, we supposed that a spiral coil *C_1_* lies on the *x*–*y* plane with the center at the origin, and coil *C_2_* is placed above *C_1_* at a distance *h* apart, as shown in Figure 1b. The direction of the *y*-axis is into the page, not shown here. The primary coil’s *C*_1_ radius is *R_A_*, the secondary coil *C*_2_ radius is *R_B_*, the distance between them is *h*, P and Q are the tangential elements on the *C*_1_ and *C*_2_, respectively, the gap between P and Q is represented by *R*, *θ*_1_ is the angle of R_A_ relative to *x*-axis of *C*_1_, and the angle of *R_B_* is *θ*_2_.

Mutual inductance of the planar spiral coil can be presented by Neumann’s equation as follows:

(1) $$M=\frac{\mu_{0}}{4\pi }\oint_{C_{1}}\oint_{C_{2}}\frac{dl_{1}dl_{2}}{R}$$

where *μ*_0_ is the vacuum permeability, *dl*_1_ and *dl*_2_ are line elements, and *R* is the separation between it. Eventually, the mutual inductance of the planar circular spiral coil can be determined by finding *dl_1_*, *dl_2_*, and *R* in Equation (1).

To estimate the mutual inductance, when the gap between the two coils is larger than wire diameter, the thick coil can be denoted by equation of planar spiral coil [23]. The equation of the circular spiral coil, motivated by the Archimedean spiral coil can be described by

(2) $$R=R_{i}+a\theta ,\theta_{i}\leq \theta \leq \theta_{o}$$

(3) θ=2πN

(4) $$a=\frac{s}{2\pi }$$

where *R_i_* is an initial radius, *N* is the number of turns, *s* is the gap between turns, *a* is pitch factor, and *θ_i_* and *θ_o_* are initial and final angle of Archimedean spiral coil. The pitch factor *a* affects the gap between turns.

The values of the coil’s parameters, such as inner radius *R_i_*, outer radius *R_o_*, number of turns *N*, and the gap between them *s*, are calculated from the spiral coil Equation (5).

(5) $$R_{o}=R_{i}+N\times s$$

The pitch factor *a* can also be determined by substituting the value of *s* in Equation (2).

Using Equation (2), the equation of *C*_1_ and *C*_2_ can be expressed as Equations (6) and (7).

(6) $$R_{A}=R_{i}{}_{1}+a_{1}\theta_{1}$$

(7) $$R_{B}=R_{i}{}_{2}+a_{2}\theta_{2}$$

Under the cylindrical coordinate system, the tangential elements *dl*_1_ and *dl*_2_ can be described as

(8) $$dl_{1}=-R_{A}\sin\theta_{1}d\theta_{1}\overset{\wedge }{x}+R_{A}\cos\theta_{1}d\theta_{1}\overset{\wedge }{y}$$

(9) $$dl_{2}=-R_{B}\sin\theta_{2}d\theta_{2}\overset{\wedge }{x}+R_{B}\cos\theta_{2}d\theta_{2}\overset{\wedge }{y}$$

where *R_A_* and *R_B_* represent the distance from the origin to the tangential elements *C*_1_ and *C*_2_, respectively.

(10) $$dl_{1}.dl_{2}=R_{A}R_{B}\cos\left(\theta_{2}-\theta_{1}\right)d\theta_{1}d\theta_{2}$$

The distance *R* between *dl*_1_ and *dl*_2_ can be denoted by using cosine law.

(11) $$\begin{array}{ll} R^{2}= & {\left(R_{i1}+a_{1}\theta_{1}\right)}^{2}+{\left(R_{i2}+a_{2}\theta_{2}\right)}^{2}\\ & -2\left(R_{i1}+a_{1}\theta_{1}\right)\left(R_{i2}+a_{2}\theta_{2}\right)\cos\left(\theta_{2}-\theta_{1}\right)+h^{2} \end{array}$$

Substituting Equations (10) and (11) in Equation (1), the mutual inductance of perfectly aligned planar spiral coil is obtained in (12).

(12) $$\begin{array}{l} M=\frac{\mu_{0}}{4\pi }\iint_{2\pi N}\frac{jd\theta_{1}d\theta_{2}}{\sqrt{{\left(R_{i1}+a_{1}\theta_{1}\right)}^{2}+{\left(R_{i2}+a_{2}\theta_{2}\right)}^{2}-2j+h^{2}}}\\ j=\left(R_{i1}+a_{1}\theta_{1}\right)\left(R_{i2}+a_{2}\theta_{2}\right)\cos\left(\theta_{2}-\theta_{1}\right) \end{array}$$

Equation (12) is a double integral from, therefore, it is hard to be represented with an elementary formula and it can be solved by numerical tool. This is a disadvantage of this method. However, maintaining the accuracy in various cases is a powerful advantage.

### 2.2. Lateral Misalignment of the Planar Spiral Coil

In most wireless power applications, the secondary coils are often laterally misaligned. The mutual inductance depends on the combined link of the magnetic field between two coils. Displacement of one coil relative to another significantly reduces the shared magnetic field and thus the mutual inductance. Therefore, it is important to consider the misalignment. 

**Figure 2:**
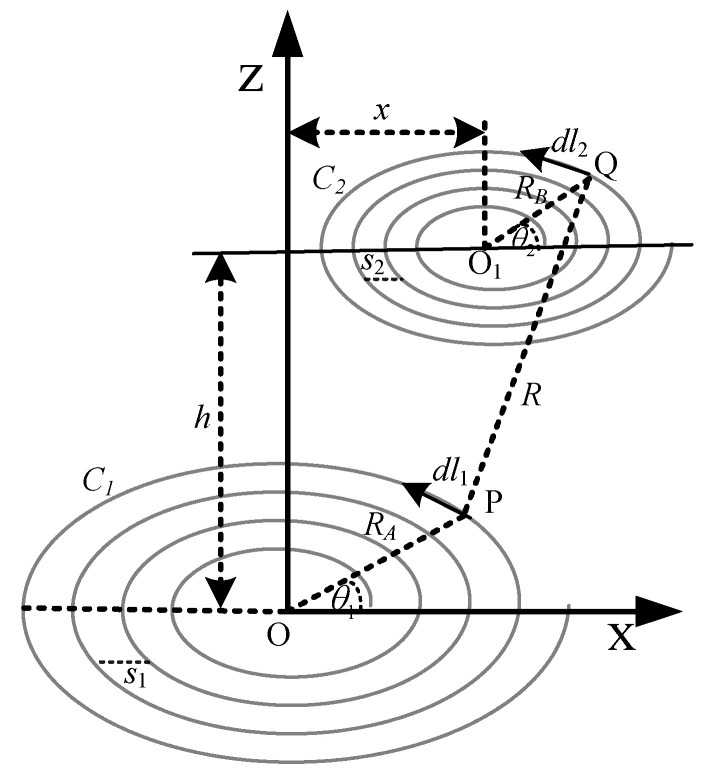
Planar spiral coil with lateral misalignment.

The arrangement of the planar spiral coil for lateral misalignment is shown in Figure 2. The secondary coil is laterally displaced towards the positive *x*-axis and denoted by the variable *x*. So, in this case, the distance between points P and Q can be obtained as Equation (13).

(13) $$\begin{array}{ll} R^{2}= & R_{A}^{2}+R_{B}^{2}+h^{2}+x^{2}-2R_{A}R_{B}\cos\left(\theta_{2}-\theta_{1}\right)\\ & +2xR_{A}\cos\theta_{1}-2xR_{B}\cos\theta_{2} \end{array}$$

The final equation of mutual inductance with lateral misalignment can be achieved by replacing the denominator of Equation (12) with the Equation (13).

(14) $$M=\frac{\mu_{0}}{4\pi }\iint_{2\pi N}\frac{jd\theta_{1}d\theta_{2}}{\sqrt{\begin{array}{l} R_{A}^{2}+R_{B}^{2}+h^{2}+x^{2}-2j\\ +2xR_{A}\cos\theta_{1}-2xR_{B}\cos\theta_{2} \end{array}}}$$

Equation (14) can be applied to calculate the mutual inductance at non-axial lateral displacement. It can be used for axial distances as well when the *x* = 0. In this case, it will be simplified to Equation (12).

## 3. Simulation Verification

To verify the simplified equation, many couples of spiral coils with different sizes, gap distance, and number of turn, are simulated on 3-D Maxwell FEM. The calculation results are compared to the simulation results and errors are calculated relative to the simulation result as well. The type of solution is Magneto static. Mesh is assigned as a length based on the coil and its boundary. The element length is the default value. The current is assigned uniformly across the coil’s cross-section for simplicity’s purpose.

In the following Tables, the mutual inductances of a number of a circular spiral coils are calculated with different gap distances s between their turns, outer radiuses Ro, and number of turns, and the results are compared to the simulation result. Ri and h represent the inner radius and distance between the primary and secondary coil respectively. Both these parameters are considered constant in the simulation comparison. FEM shows the simulation result.

The above Tables validate the simplified mutual inductance equation relative to simulation results for all cases. Table 1 shows, smaller the gap between the turn *s*, lesser will be the error. When the outer radius *R_o_* and the number of turns *N* increased, the error increased slightly, as indicated in Table 2 and Table 3, respectively. However, for all cases the errors are below 4% which is in acceptable range. Thus, it proves the accuracy of the simplified equation.

## 4. Experimental Verification

Wireless power technique has been employed in wide power applications, ranging from low power electric shaver, smartphone charger, and biomedical implant devices to medium power drone chargers and large power electric cars. Therefore, it is important to validate the simplified mutual equations for different geometry of the spiral coils. Depending on the application, we have constructed two small sample coils with turn number 10 and two large coils with the number of turn 16. The larger size of the coil is limited to 170 mm in diameter due to the capacity of a 3D printer which is used to make a bobbin for winding the wire. The PLA (polylactic acid) filament is adopted for constructing bobbins. These coils are sample examples for different wireless applications. The arrangement for determining the mutual inductance of the spiral coils with different axial and non-axial distances are as follows. Firstly, inductances are calculated between two large primary and large secondary coils; secondly, between small primary and small secondary; and finally, between large primary and large secondary. The calculation results are compared both by simulation and experiment.

**Figure 3:**
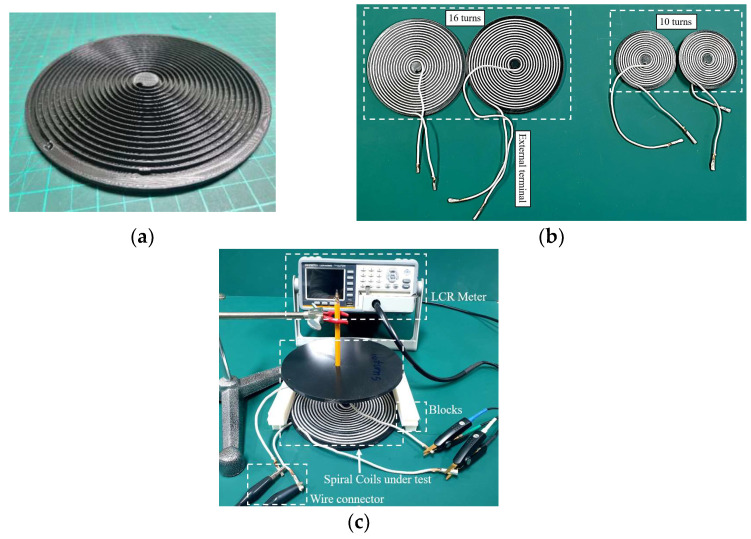
Experimental setup for measuring mutual inductance. (**a**) Bobbin. (**b**) Spiral coil with bobbin. (**c**) Measuring components.

Figure 3a,b show the bobbins and bobbins with spiral winding and Figure 3c describes the experimental setup for three different coil configurations. Special blocks with each 5 mm thickness are designed in the 3D printer for measuring the gap between the primary and secondary coil in the axial and non-axial cases. The stand is used to hold the secondary coil with the help of a pencil to show the coupling distance. A steel ruler and Vernier caliper are utilized to maintaining the accuracy of axial and non-axial distances.

The structural parameters of the sample coils are shown in Table 4 and categorized into the small and the larger coil. Where *N* is the turn number, *s* is the gap between turns, R_i_ is the inner radius of the planar spiral coil, and *R_o_* is the outer radius.

### 4.1. Selection of Wire and Its Measurement

We have used Litz wire for fabricating the spiral coil due to its vast advantages over solid wires. Litz wire has lower AC resistance than a solid conductor at higher frequencies. In a solid conductor, the current density is higher at its surface and exponentially decreases towards the center due to an effect called the skin effect. As a result, it decreases the effective cross-sectional area of the wire and, hence, increases the resistance. On the other hand, Litz wire is made of thin strands insulated with each other and twisted in a manner that, at some point, each strand holds a position in the cross-sectional area [24]. The thinning and twisting of the strand help to mitigate the skin and proximity effect, respectively. The proximity effect is caused by the current of nearby strands in the bundle, by the leakage fluxes from air gaps and core, and by the external field from other conductors [25].

In this study, the Litz wire with 500 strands and 0.12 mm diameter is chosen. The total diameter of Litz wire is 3.6 mm, and it is decided empirically by Equation (15).

(15) De=1.6((Ds2)2Ns)2

where *Ns* is the total number of strands, *Ds* is the diameter of one strand, and *De* is the estimated diameter of Litz wire [26].

The length of the wire is also one of the important parameters which influence the inductance and the *DC* resistance. Therefore, the accurate length and the resistance can be determined from Equations (16) and (17).

(16) $$L_{total}=\int_{0}^{2\pi N}\sqrt{R_{o}^{2}+{\left(\frac{dR_{o}}{d\theta }\right)}^{2}d\theta }$$

(17) $$R_{DC}=\frac{l(\mathrm{mm})}{\sigma n\pi r^{2}}$$

where *l* the length of the wire, *σ* is constant and its values is 58,000, *n* is the number of strands, and *r* is the radius of one strand.

Soldering quality is another factor that affects *DC* resistance. Good quality lead and specific pot temperature are required for high-quality soldering. Thus, a proper soldering set and particular jig were prepared for this purpose.

### 4.2. Mutual Inductance Measurement Method

**Figure 4:**
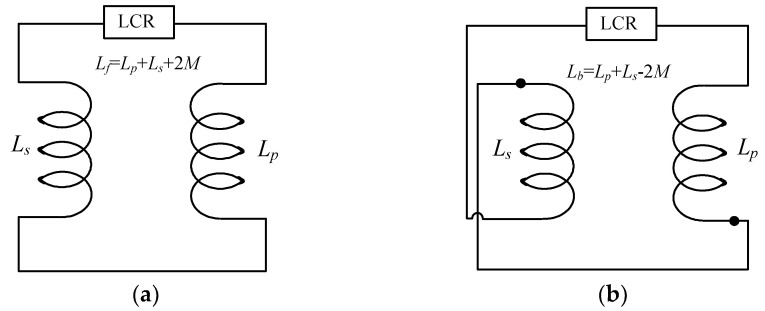
Two different connection mode for measuring mutual inductance. (**a**) Forward mode. (**b**) Backward mode.

The measurement of mutual inductance is performed by a Gwinstek LCR-6200, and their results are compared with the calculated and simulated results. The following method is used to measure the mutual inductance. Firstly, the inductance is measured when the primary and secondary coil is connected in forwarding mode, as shown in Figure 4a. In this mode, the inductance would be equal to the summation of the primary and secondary inductances and twice the mutual inductance. Secondly, the primary and secondary coils are connected in a backward mode, as shown in Figure 4b. In this mode, the inductance would be the sum of the inductances of each primary and secondary coil minus twice the mutual inductance Finally, the actual mutual inductance is obtained by subtracting the backward inductance from the forward connection inductance and dividing it by 4, as shown in Equation (18).

(18) $$M=\frac{L_{f}-L_{b}}{4}$$

The operating frequency is selected 20 kHz to ignore the skin effect.

### 4.3. Mutual Inductances of Different Spiral Coil Configurations for Aligned Distances and Their Error Comparison

Considering the wireless charging applications, firstly, the mutual inductance between two large sample axial coils under the condition of increasing axial distance *h* is measured. The measured results are compared to the calculation and the simulation results. Some emerging applications, like transthermic wireless power transfer systems, require a small secondary coil compared to the primary coil [27].

**Figure 5:**
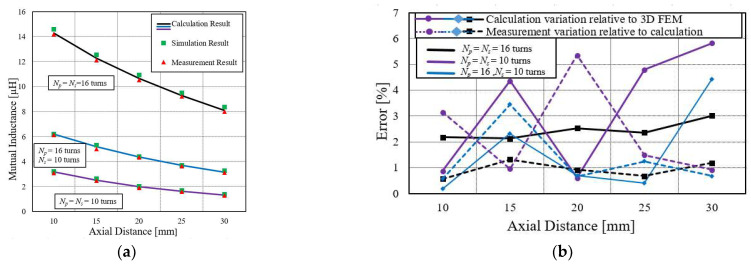
Mutual inductance. (**a**) Comparison between different coils configuration at various axial distances. (**b**) Error comparison of coils relative to calculation and FEM.

To validate the simplified equation for these kinds of systems, mutual inductance between large primary and small secondary coils are also measured for various axial distances. Finally, inductances between two small primary and small secondary coils are measured. Their comparison results are shown in Figure 5 where *N_p_* and *N_s_* denote the number of turns of primary coil and secondary coil, respectively.

The calculated results of the large, medium, and small configurations of coils are denoted by black, blue, and purple lines. The green square box shows the simulation results, and the red triangle box represents the measurement result. Figure 5a shows that the mutual inductance decreases with the increase in the axial distance, as can be described by the Faradays laws of magnetic induction, which state that the mutual inductance between two coils depends on the shared coupling of magnetic. The smaller the axial distance, the stronger the magnetic flux, and, thus, the higher the mutual inductance. The magnetic flux linkage would be weaker as the axial distance between coils increases. Therefore, this would cause the deceases in the mutual inductance. 

If the axial distance keeps increasing, a point is reached where the mutual inductance would be zero, and all the lines will converge at the common point. Thus, to maintain receiving the power of WPT at the required level, it is necessary to keep the secondary coil at a certain optimized distance. Figure 5b is the error comparison of calculation results relative to simulation and measurement results. It shows that the error for the large coil is small, under 3%, compared to the small coil error, which is less than 9%. Thus, the comparison of simulation and measurement results with the calculated one certifies the accuracy of the simplified method.

### 4.4. Behavior of Mutual Inductance between Two Large Primary and Large Secondary Spiral Coils under Lateral Misalignment and Their Error Comparison

In some power transfer applications, such as biomedical implants and drone charging systems, the secondary coil is often misplaced. As a result, mutual inductance is drastically affected, and, therefore, so is the power link performance. This misaligned effect is important to be investigated. This case has shown, in Figure 2, that the secondary coil is laterally displaced. Regarding the applications perspective, using Equation (14), mutual inductance is calculated at discrete lateral misaligned distances for three different coil configurations: large primary and large secondary coil; small primary and small secondary; and the small secondary, large primary coil.

**Figure 6:**
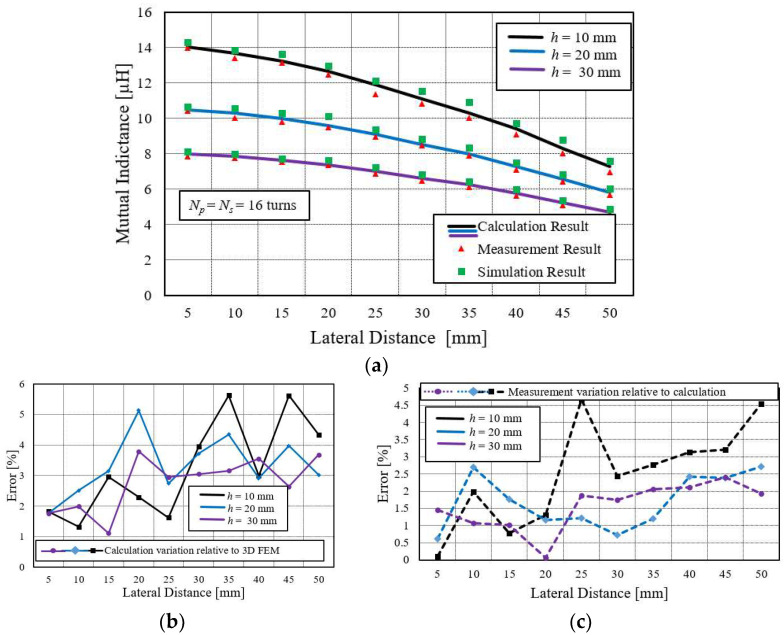
(**a**) Mutual inductance comparison between large primary and large secondary coils at various lateral misalignment distances. (**b**) Calculation variation relative to simulation (FEM) for *N* = 16. (**c**) Measurement variation relative to calculation.

The calculated results are compared with simulation and measurement results to verify its correctness. The comparison details are represented in Figure 6a. In Figure 6a, the comparison between two large coils is shown. The calculated results are indicated with the black, blue, and purple lines. The green square box and a red triangle box represent the simulation and measurement results. Figure 6b shows the calculation variation relative to 3D FEM, while Figure 6c represents the variation of measurement result to the calculation result. In the configuration of this large coil, the variations are below 6% for all cases.

#### 4.4.1. Behavior of Mutual Inductance between Two Small Primary and Small Secondary Spiral Coils under Lateral Misalignment and Their Error Comparison

**Figure 7:**
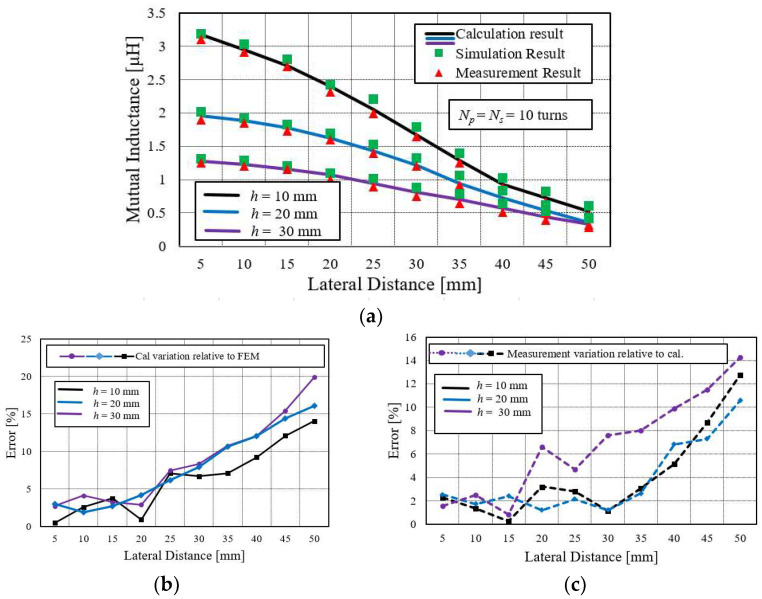
(**a**) Mutual inductance comparison between small primary and small secondary coils at various lateral misalignment distances. (**b**) Calculation variation relative to simulation (FEM) for *N* = 10. (**c**) Measurement variation relative to calculation.

Figure 7a shows the variation of mutual inductance for the lateral misalignment at various coupling distances for small primary and small secondary spiral coils. Their comparative error analysis is represented in Figure 7b,c. It is observed that the mutual inductance declines faster at small h compared to larger *h*. Moreover, it can be seen that the error for the small turn of the spiral coil (*N* = 10) increases gradually when the lateral displacement is more than the maximum radius of the coil. The variations of the calculation result to the FEM shows more than 20%, while measurement variation relative to the calculation is above 15%. Therefore, the working region is set as the outer radius of the coil for the lateral displacement.

Furthermore, such a big lateral displacement is not required in most wireless power applications because the mutual inductance also approaches zero in such large lateral displacement.

#### 4.4.2. Behavior of Mutual Inductance between Large Primary and Small Secondary Spiral Coils under Lateral Misalignment and Their Error Comparison

Mutual inductances are calculated at three different axial distances: *h* = 10 mm, 20 mm, and 30 mm, and at various lateral distances, ranging from 0 to 50 mm with a 5 mm gap distance difference between them. The result of mutual inductance at each axial gap is differentiated with a unique color line.

Lateral misalignment of the coil also drastically affects the mutual inductance. This effect is higher at the larger misalignment than at the small one. As the misplaced distance increases, the mutual inductance reduces. This reduction of the *M* is due to the lower influence of the magnetic flux distribution of the primary coil on the displaced coil.

**Figure 8:**
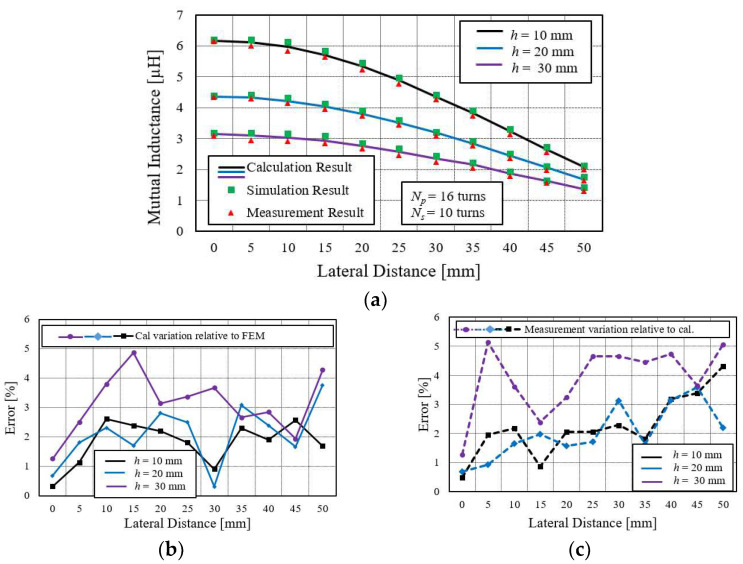
Mutual inductance comparison between large primary and small secondary coils at various lateral misalignment distances. (**a**) Primary large and secondary small coil. (**b**) Calculation variation relative to simulation (FEM) for *N_p_* = 16, *N_s_* = 10. (**c**) Measurement variation relative to calculation.

The detail of a large primary and a small secondary coil is illustrated in Figure 8a, while its error comparison is shown in Figure 8b,c. In this case, the calculation error relative to FEM and measurement result is less than 6%, which verifies the accuracy of the simplified method.

The mutual inductances of all coil configurations will meet at a common point if the lateral distance increases beyond a certain limit. The limit is the outer radius of the coil. The mutual inductance approaches zero when the lateral misaligned distance increases beyond the highest radius of the coil, and the simplified equation is not accurate under this condition. However, such a higher lateral distance may not require in practice. The maximum working region for lateral misalignment is less than the radial length of the coil.

Mutual inductance values in Figure 8a show that the curve decline rate is higher when the misplaced distance is increased compared to the smaller displacement, which indicates a flat curve. Thus, it can be concluded that the lateral misalignment should be little for obtaining consistent mutual inductance value. It can be described by the magnetic field theory, which states that the concentration of flux is higher at the center of the coil, and it gradually becomes weaker as it moves away from the center.

It is also observed from Figure 6a, Figure 7a, and Figure 8a that a decline in the rate of mutual inductance at higher axial distance is slightly lower than at the lower axial distance *h*. It can be easily justified by the field distribution of spiral coil. When the electric current flows into the coil, the field is generated and distributed mainly in two directions: radial direction, and vertical direction. Since the radial field has a negligible influence on mutual inductance, it can be ignored. The concentration of the vertical field is higher near the coil than at a distance away from it. Therefore, when the secondary coil moves away from the center of the primary coil at a lower axial distance, it cuts more flux line compared to coil at higher h where flux lines are fewer. It causes slightly more decline of *M* [28].

Thus, these comparisons for different coil configurations prove the accuracy of the simplified mutual inductance equation for all cases.

### 4.5. Coupling Coefficient of Spiral Coil

For wireless power applications, one of the parameters which determine the maximum output power is the coupling coefficient, and it can have a huge impact on the system efficiency [29].

**Figure 9:**
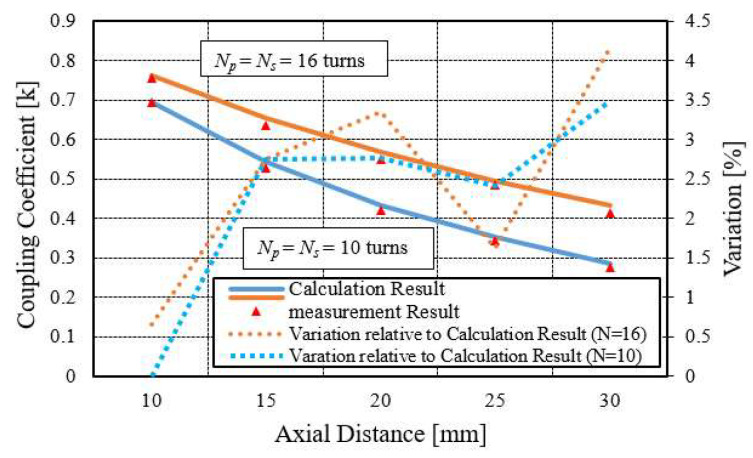
Coupling coefficient as a function of axial distance.

The simplified mutual equation has also verified by the coupling coefficient. It is the fraction of the total flux flowing from one coil to another. Its value ranges from 0 to 1 and is represented by *k*. For perfectly coupled coil (whole flux produced by one coil link another coil), the value of *k* = 1, while *k* > 0.5 shows the tightly coupled and the loosely coupled coils can be described by *k* < 0.5. It depends on the physical configuration of the coil, such as orientation, windings, core, and the axial distance between them. *k* can defined by Equation (19).

(19) $$k=\sqrt{\frac{M^{2}}{L_{p}L_{s}}}$$

where *M* is the mutual inductance, *L_p_* is the self-inductance of primary coil, and *L_s_* is the self-inductance of secondary coil. Self-inductance of the coil can be found by replacing the axial distance parameter *h* in the denominator of Equation (12) with the wire diameter w. Using a modified Equation (12), the self-inductance of the sampled 10 turns and 16 turns spiral coil were 4.584 µH and 18.734 µH, respectively. It can also be measured by an LCR meter. The measured self-inductance values are 4.712 µH and 19.310 µH, alternately. *k* relative of axial distance is depicted in Figure 9.

The minimum distance is 10 mm, and the maximum is 30 mm. The difference between successive *h* changes is 5 mm. The calculated coupling coefficient values is represented with the orange and blue lines, while the measured value is indicated by the small red triangular box.

The comparison result shows good agreement between measured and calculated values, which is below 5%. It can be seen that with the increase in the axial distance, the k reduces to zero, which is clear from the definition of the k that the magnetic flux linkage of one coil regarding another coil drops as the distance between them increases. Figure 9 also indicates that for the fixed axial distance, the value of k is a little smaller for the small coil compared to the large coil due to the higher magnetic flux linkage in the large coil than the small coil.

## 5. Conclusions

In this paper, a simplified and easy equation of mutual inductance for two coaxial and non-coaxial circular planar spiral coils has been presented. The Neumann formula has been adopted to derive this equation. Each step of mathematical derivation has been given. Compared to the conventional method, this simplified method reduces the calculation complexity and long numerical computations. The comparison results have shown that the error is larger for a spiral coil with a small number of turns. For the same axial distance, the error is below 9% for smaller coils while it is less than 3% for the larger spiral coil. And in the case of non-axial distance, the error increases when the lateral misaligned distance is greater than the maximum radius of the spiral coil. However, such a big misalignment is not required practically because the mutual inductance is approaching zero in such a case.

The calculation result has been compared by simulation for different geometry, either sparsely wounded or densely wounded. The comparison of the simulation result confirms the simplified equation’s correctness with a difference of only 4%. Secondly, it has been compared with the simulation and the measurement result at various axial and non-axial distances for different coil categories, such as between small primary and small secondary coil, large primary and small secondary coil, and large primary and large secondary coil. This comparison also certifies the simplified equation accuracy. The final validity comparison has concluded between calculated and measured values of the coupling coefficient relative to axial distance. The error between them is under 5%. Thus, these comparisons have shown the correctness of the simplified mutual inductance equation, which makes it a suitable candidate to be adopted in different wireless power applications like biomedical implants, wireless charging systems, and contactless battery charging.

In this work, mutual inductance was derived by supposing the uniform current flow in the coil. However, the inductance value could be different for un-uniform current flow. It requires further research in this regard. Furthermore, the simplified mutual inductance equation is in double integral form. It can be simplified to a single integral form, which will be considered in future work.

## Figures and Tables

**Table 1:** Comparison result with variation in gap between the turn.

Parameters				Results		
*h* (mm)	*s* (mm)	*R_i_* (mm)	*R_o_* (mm)	FEM (µH)	(11) (µH)	Error (%)
10	7.5	10	85	5.85	5.67	3.07
10	6.25	10	85	8.25	8.03	2.66
10	5.35	10	85	11.24	11.00	2.13
10	4.68	10	85	14.56	14.29	1.85

**Table 2:** Comparison result with variation in outer radius.

Parameters				Results		
*h* (mm)	*N* (mm)	*R_i_* (mm)	*R_o_* (mm)	FEM (µH)	(11) (µH)	Error (%)
10	10	10	85	5.85	5.67	3.07
10	10	10	95	6.61	6.40	3.17
10	10	10	105	7.39	7.15	3.24
10	10	10	115	8.19	7.91	3.41

**Table 3:** Comparison result with variation in number of turns.

Parameters				Results		
*h* (mm)	*N* (mm)	*R_i_* (mm)	*s* (mm)	FEM (µH)	(11) (µH)	Error (%)
10	10	10	7.5	5.85	5.67	3.07
10	12	10	7.5	9.87	9.56	3.14
10	14	10	7.5	15.86	15.29	3.59
10	16	10	7.5	23.48	22.67	3.44

**Table 4:** Geometrical parameters of experimental coils.

Coil Size	*N* (mm)	*R_i_* (mm)	*s* (mm)	*R_o_* (mm)
Small Coil	10	10	4	50
Large Coil	16	10	4.68	85

## Data Availability

Not Applicable.
